# Circ_0000215 Exerts Oncogenic Function in Nasopharyngeal Carcinoma by Targeting miR-512-5p

**DOI:** 10.3389/fcell.2021.688873

**Published:** 2021-10-26

**Authors:** Xinping Chen, Weihua Xu, Zhichao Ma, Juan Zhu, Junjie Hu, Xiaojuan Li, Shengmiao Fu

**Affiliations:** ^1^Department of Central Laboratory, Hainan General Hospital, Hainan Hospital Affiliated to The Hainan Medical College, Haikou, China; ^2^Hainan Provincial Key Laboratory of Cell and Molecular Genetic Translational Medicine, Haikou, China

**Keywords:** circ_0000215, nasopharyngeal carcinoma, miR-512-5p, PIK3R1, oncology

## Abstract

**Background:** Increasing circular RNAs (circRNAs) are reported to participate in cancer progression. Nonetheless, the role of circRNAs in nasopharyngeal carcinoma (NPC) has not been fully clarified. This work is aimed to probe the role of circ_0000215 in NPC.

**Methods:** Circ_0000215 expression in NPC tissues and cell lines was examined by quantitative real-time polymerase chain reaction (qRT-PCR). Cell counting kit-8 (CCK-8) assay, 5-bromo-2′-deoxyuridine (BrdU) assay, scratch healing assay and Transwell experiment were executed to investigate the regulatory function of circ_0000215 on the proliferation, migration and invasion of NPC cells. RNA immunoprecipitation (RIP), pull-down and dual-luciferase reporter experiments were utilized to determine the binding relationship between circ_0000215 and miR-512-5p, and between miR-512-5p and phosphoinositide-3-kinase regulatory subunit 1 (PIK3R1) 3′UTR. The effects of circ_0000215 on NPC growth and metastasis *in vivo* were examined with nude mice model. Western blot was applied to detect the regulatory effects of circ_0000215 and miR-512-5p on PIK3R1 expression.

**Results:** Circ_0000215 was overexpressed in NPC tissues and cell lines. The functional experiments confirmed that knockdown of circ_0000215 impeded the growth and metastasis of NPC cells *in vitro* and *in vivo*. Additionally, circ_0000215 could also work as a molecular sponge to repress miR-512-5p expression. PIK3R1 was validated as a target gene of miR-512-5p, and circ_0000215 could increase the expression level of PIK3R1 in NPC cells via suppressing miR-512-5p.

**Conclusion:** Circ_0000215 is overexpressed in NPC and exerts oncogenic effects in NPC through regulating miR-512-5p/PIK3R1 axis.

## Introduction

Nasopharyngeal carcinoma (NPC) is a malignant epithelial neoplasm of the nasopharynx, and EBV infection is a known risk factor associated with NPC tumorigenesis and development (Global Cancer Observatory, 2018; Chen et al., 2019). NPC is considered to be sensitive to radiation, and radiotherapy-based comprehensive treatment is the main strategy to treat this disease (Tham and Lu, 2010; Xiao et al., 2011). However, the number of patients dying from NPC is increasing worldwide, and for the patients with metastasis, the prognosis is poor (Bray et al., 2018). Therefore, it is crucial to develop better diagnostic and therapeutic approaches dependent on the understanding of the molecular mechanism of NPC progression.

Non-coding RNAs (ncRNAs) have attracted a lot of attention in cancer research in recent years (Wilusz and Sharp, 2013; Skvortsova et al., 2018). Circular RNA (circRNAs) are RNA transcripts with a covalent circular conformation formed by direct reverse splicing or exon skipping of precursor mRNAs. CircRNAs are stable and resistant to RNase, and their structures have 5′–3′ polarity (Chen, 2016). An increasing amount of evidence suggests that circRNAs are implicated in tumorigenesis and cancer progression by modulating important biological processes such as cell growth, migration, differentiation, drug resistance, metabolism, and so on (Hua et al., 2019; Kristensen et al., 2019; Yu et al., 2019). For instance, circ_0000096 modulates the growth and migration of gastric cancer cells by modulating cyclin D1, CDK-6, MMP-2, MMP-9, and E-cadherin expressions (Li et al., 2017). Reportedly, circ-ITCH restrains bladder cancer progression by sponging miR-17/miR-224 to modulate p21 and PTEN expressions (Yang et al., 2018). The novel protein encoded by circ-PPP1R12A accelerates colon carcinogenesis and metastasis through Hippo-YAP signaling (Zheng et al., 2019). Nevertheless, biological functions and underlying mechanisms of circRNAs in NPC are still largely unknown.

Several possible mechanisms by which circRNAs exerts their biological functions have been proposed. For instance, circRNAs can work as competitive endogenous RNAs (ceRNAs) that competitively bind with miRNAs, and circRNA can function as bait or scaffold to interact with RNA-binding proteins (RBPs), to regulate the sub-cellular localization, assembly and activity of proteins (Hansen et al., 2013; Li Y. et al., 2015; Ebbesen et al., 2016; Du et al., 2017; Pamudurti et al., 2017). In this work, we report that hsa_circ_0000215 [a circRNA derived from intron 2 and exons 3 of the calcium/calmodulin dependent protein kinase ID (CAMK1D), circ_0000215], was up-regulated in NPC tissue. The knockdown of circ_0000215 remarkably represses the proliferation, migration, invasion, and epithelial-mesenchymal transition (EMT) of NPC cells, via functioning as a ceRNA to regulate miR-512-5p and phosphoinositide-3-kinase regulatory subunit 1 (PIK3R1).

## Materials and Methods

### Patients and Specimens

Thirty two pairs of NPC tissues and adjacent normal tissues were obtained from the patients (18 male and 14 female, aged 28–71 years with a mean age of 48.3 years) who received biopsy at Hainan General Hospital from 2018 to 2020. All tumor tissues and paired normal tissues were confirmed by experienced pathologists. None of the patients received radiotherapy or chemotherapy before biopsy. Patients diagnosed with infectious diseases, autoimmune diseases, or other malignant tumors were excluded. The clinicopathological features of the patients were listed in Table 1. Written informed consent was obtained from all subjects. This work was endorsed by the Ethics Committee of Hainan General Hospital.

**TABLE 1:** Correlation between circ_0000215 expression and the clinicopathological characteristics of 32 patients.

**Clinical characteristics**	**Total (*n* = 32)**	**Circ_0000215 expression**		***P*-value**
		**Low (16)**	**High (16)**	
**Age (years)**				
≤ 50	19	10	9	0.719
>50	13	6	7	
**Gender**				
Male	18	8	10	0.476
Female	14	8	6	
**T stage**				
T1+T2	11	7	4	0.458
T3+T4	21	9	12	
**Lymph node metastasis**				
With	15	4	11	0.032*
without	17	12	5	
**Clinical stage**				
I–II	13	3	10	0.029*
III–IV	19	13	6	

Chi-squared test was utilized to analyze clinical data. *P < 0.05.

### Cell Lines and Cell Culture

HONE1 is an EBV-negative cell line, which was established from a biopsy specimen from a poorly differentiated squamous cell carcinoma of the nasopharynx. 5–8F is an EBV-negative NPC cell line, which is derived from metastatic nodules with highly tumorigenic and metastatic potential. SUNE-1 is an EBV-negative NPC cells, which has high metastatic potential. CNE-2 cell line is derived from poorly differentiated squamous cell carcinoma and it is an EBV-negative NPC cell line. C666-1 is an undifferentiated and EBV-positive NPC cell line. These cell lines and the immortalized normal nasopharyngeal epithelial cell line NP69 were available from American Type Culture Collection (ATCC, Manassas, VA, United States). All cell lines were cultured in Dulbecco’s modified Eagle’s medium (DMEM) (Gibco, Grand Island, NY, United States) containing 10% fetal bovine serum (FBS) (Gibco, Grand Island, NY, United States) at 37 °C in 5% CO_2_.

### Quantitative Real-Time Polymerase Chain Reaction

Total RNA was extracted from tissues and cells using TRIzol reagent (Invitrogen, Carlsbad, CA, United States). Total RNA was reverse transcribed into complementary DNA (cDNA) using a PrimeScript^TM^ RT Kit (Takara, Dalian, China). qRT-PCR was implemented with TaqMan Universal PCR Master Mix (Applied Biosystems, Carlsbad, CA, United States) in AB7300 thermo-recycler (Applied Biosystems, Carlsbad, CA, United States). Glyceraldehyde 3-phosphate dehydrogenase (GAPDH) and β-actin were used as an endogenous control for circ_0000215, CAMK1D and PIK3R1, and U6 and U48 were employed as the endogenous controls for miR-512-5p expression level. 2^–ΔΔCt^ method was used to calculated the relative expression, in which ΔΔCT = (C_T_ target gene—C_T_ endogenous control) test—(C_T_ gene—C_T_ endogenous control) control. The specific primers were obtained from GenePharma (Shanghai, China) and the primer sequences are as follows: for circ_0000215, 5′-TGCAGAATGGCTTAGAACACC-3′ (forward) and 5′-TTCA GAAGTTGCCGTGAATG-3′ (reverse); for CAMK1D, 5′-CAT AGGACTGGAAGACCGAAGTTTT-3′ (forward) and 5′-CTCG AGTCAGTACAGTTTGTGAGAA-3′ (reverse); for PIK3R1, 5′-AAGAAGTTGAACGAGTGGTTGG-3′ (forward) and 5′-G CCCTGTTTACTGCTCTCCC-3′ (reverse); for GAPDH, 5′-CTT AGATTTGGTCGTATTGG-3′ (forward) and 5′-GAAGATG GTGATGGGATT-3′ (reverse); for β-actin, 5′-AGGGAAAT CGTGCGTGAC-3′ (forward) and 5′-CGCTCATTGCCGAT AGTG-3′ (reverse); for miR-512-5p, 5′-TCGAGTCCCTCAC TGTTACCCTTG-3′ (forward) and 5′-TAGATGACTTAA GCCTCAGCAGCA-3′ (reverse); for U6, 5′-CTCGCTTCGG CAGCACA-3′ (forward) and 5′-AACGCTTCACGAATTTGC GT-3′ (reverse); for U48, 5′- AGTGATGATGACCCCAGG TA-3′ (forward) and 5′-GGTCAGAGCGCTGCGGTGA T-3′ (reverse). The subcellular localization of circ_0000215 was determined by qRT-PCR, after the RNA in nucleus and cytoplasmic fractions of NPC cells were isolated by a PARIS^TM^ Kit (Ambion, Life Technologies, United States). U6 snRNA and GAPDH were employed as positive controls for the nuclear and cytoplasmic fractions, respectively.

### Actinomycin D and RNase R Treatment

To block transcription, 2 mg/ml Actinomycin D (Sigma-Aldrich, St. Louis, MO, United States) was used to treat the cells. For RNase R treatment, 5 μg of total RNA was incubated with 3 U/μg RNase R (Epicenter Technologies, Madison, WI, United States) at 37°C for 15 min. After the treatment with Actinomycin D or RNase R, circ_0000215 and CAMK1D mRNA expression were examined by qRT-PCR.

### Cell Transfection

100 nM circ_0000215 small interfering RNA (si-circ_0000215#1/#2), PIK3R1 small interfering RNA (si-PIK3R1) or siRNA negative control (si-NC), 50 nM miR-512-5p mimic, miR-512-5p inhibitor and the corresponding negative control, 100 nM circ_0000215 overexpression plasmids or empty plasmids were transfected into HONE1 and CNE-2 cells, respectively, with Lipofectamine^®^ 3000 (Invitrogen, Carlsbad, CA, United States). The plasmids and oligonucleotides were designed and synthesized by GenePharma (Shanghai, China). To validate the transfection efficiency, 36 h after the transfection, qRT-PCR was performed.

### Cell Counting Kit-8 Experiment

Cell viability experiments were conducted using the CCK-8 kit (Dojindo Laboratories, Kumamoto, Japan). Briefly, approximately 1 × 10^3^ cells were planted into each well of 96-well plates and cultured, and then incubated with 10 μL of CCK-8 reagent at 0, 24, 48, 72, and 96 h. After 2 h of incubation, a microplate reader was used to read the optical density values at 450 nm.

### 5-Bromo-2′-Deoxyuridine Experiment

The cells inoculated in 96-well plates were cultured for 24 h, and then incubated with 10 μM BrdU solution (BD Pharmingen, San Diego, CA, United States) for 12 h, fixed with 4% paraformaldehyde for 30 min and then incubated with anti-BrdU antibody (1:1000, Sigma-Aldrich, St. Louis, MO, United States) for 1 h, and IgG was used as the negative control. Moreover, the cells were incubated with secondary antibodies (1:500, Beyotime, Shanghai, China) for 1 h. Subsequently, the cells were stained with DAPI staining solution (Beyotime, Shanghai, China). After the cells were washed by phosphate buffer saline (PBS), the number of BrdU-positive cells in the three fields of view was randomly counted under the microscope, and the percentage of BrdU-positive cells was calculated.

### Wound Healing Experiment

The NPC cells were positioned in 6-well plates, and cultured until the confluency reached 90%. The monolayer cells were scraped with a 200 μL pipette tip to make a wound, then the cells were cultured with serum-free medium. The width of the wound was measured under a microscopy at 0 and 24 h.

### Transwell Assay

NPC cells were planted in each Transwell chamber (Millipore, Billerica, MA, United States) containing 200 μL of serum-free DMEM (5 × 10^5^ cells/well). The lower compartment was filled with DMEM containing 10% FBS. The cells were cultured for 24 h, and the cells remaining on the upper surface of the filter membrane were removed, and the cells on the below surface of the filter membrane were fixed with methanol, stained with crystal violet solution, and photographed with a microscope. Matrigel (BD Biosciences, San Jose, CA, United States) was used to cover the filter in invasion assay, but not used in migration assay.

### Dual-Luciferase Reporter Experiment

The circ_0000215/PIK3R1 3′UTR sequence containing the miR-512-5p binding site was amplified and inserted into pmirGLO vectors (Promega, Madison, WI, United States) to obtain the wild type (WT) reporters, namely circ_0000215-WT, and PIK3R1-WT. Meanwhile, the mutant type (MUT) circ_0000215/PIK3R1 sequence was inserted into the empty luciferase reporter vector to obtain the circ_0000215-MUT and PIK3R1-MUT. Circ_0000215-WT/PIK3R1-WT or circ_0000215-MUT/PIK3R1-MUT were co-transfected with miR-512-5p mimic or control miRNA in HONE1 and CNE-2 cells, respectively. Finally, the relative luciferase activity of each group was measured using the Dual-Luciferase Reporter Assay System (Promega, Madison, WI, United States).

### RNA Immunoprecipitation

RIP was executed using the EZMagna RIP kit (Millipore, Billerica, MA, United States) adhered to the protocols. Briefly, HONE1, and CNE-2 cells were scraped from the plate and lysed in RIP lysis buffer. The lysates were then added into RIP buffer containing anti-Ago2 antibody or IgG (Millipore, Billerica, MA, United States), which was coupled with magnetic bead, and incubated. Proteinase K and DNase (Beyotime, Shanghai, China) were used to remove the proteins and DNA in the immunoprecipitate. Ultimately, the purified RNA was analyzed by qRT-PCR.

### RNA Pull-Down Experiment

Pierce Magnetic RNA–Protein Pull-Down Kit (Thermo Fisher Scientific, Waltham, MA) was applied for RNA pull-down assay. Briefly, WT and MUT miR-512-5p sequences were synthesized and biotinylated to get Bio-miR-512-5p-WT and Bio-miR-512-5p-MUT. The biotinylated miRNA was then transfected into HONE1 and CNE-2 cells. Then the cells were lysed on ice for 10 min in the lysis buffer (Sigma, St. Louis, MO, United States), then the lysates were centrifugated, and the supernatant was incubated with M-280 streptavidin magnetic beads overnight. Subsequently, the complex was eluted, and the RNA in the complex was extracted, and then qRT-PCR was executed to examine RNA levels.

### Western Blotting Assay

NPC cells were lysed in RIPA lysis buffer (Meilun Biotechnology Co., Ltd., Dalian, China) containing protease inhibitor cocktails (Fudebio, Hangzhou, China). Then, total protein from different samples (30 μg/per lane) was separated by SDS-PAGE and transferred onto 0.22 μM PVDF membranes (Amersham Bioscience, Piscataway, NJ, United States). After that, the membranes were blocked with 5% skimmed milk in TBST for 1 h and incubated with specific primary antibody overnight at 4°C. After being washed with TBST, the membranes were incubated with the secondary antibody for 1 h at room temperature. Next, the protein bands were developed using an enhanced chemiluminescence kit (FD8030, Fudebio, Hangzhou, China). Antibodies were listed: anti-E-cadherin (Abcam, ab76319, 1:2,000), anti-Vimentin (Abcam, ab92547, 1:2,000), anti-PIK3R1 (Abcam, ab191606, 1:1,000), anti-ERBB2 (Abcam, ab237715, 1:1,000), anti-GAPDH (Abcam, ab8245, 1:3,000), goat anti-mouse IgG H&L (HRP) (Abcam, ab205719, 1:5000), and goat anti-rabbit IgG H&L (HRP) (Abcam, ab205718, 1:5,000).

### Animal Experiments

The animal experiments followed the “Guidelines for Reporting Animal Research: *In Vivo* Experiments” and were endorsed by the Animal Care and Use Committee of Hainan General Hospital. Four-week-old male BALB/c nude mice were procured from the Animal Resources Laboratory, Chinese Academy of Sciences (Beijing, China). All mice were housed in laminar flow cabinets, free of specific pathogens, with a 12 h/12 h light/dark cycle and readily available food and water. HONE1 cells (1 × 10^6^) with stable circ_0000215 knockdown were constructed with lentivirus carrying shRNA sequence targeting circ_0000215 (HanBio, Shanghai, China). The cells were injected subcutaneously into the right side of the nude mice for subcutaneous tumor growth assays, or intravenously into the tail vein of the mice to construct lung metastasis models (10 per group). The formula for calculating the tumor volume was: length × width^2^ × 0.5. After 4 weeks, the mice were sacrificed for subsequent assays. To detect the metastatic nodules in lung, the tissue sections were deparaffinized and rehydrated, and then hematoxylin-eosin (HE) staining was performed. Histopathological changes in mouse lungs were observed under an inverted microscope: severe metastasis: the metastases ≥ 50% of the observer’s field of view; moderate metastasis: 25–50% area of the observer’s field of view was the metastatic nodules; mild metastasis: < 25% of the observer’s field of view.

### Statistical Analysis

All the experiments were executed at least in triplicate. Statistical analysis was conducted using SPSS version 19.0 software (SPSS, Inc., Chicago, IL, United States). All measurement data were presented as means ± standard deviation (SD). Significance of the difference was evaluated by Student’s *t*-test for two groups and by one-way ANOVA with *post-hoc* test for more than two groups. The correlations were measured by Pearson correlation’s coefficient. The differences were considered to be statistically significant if *P* < 0.05.

## Results

### Circ_0000215 Expression Is Up-Modulated in Nasopharyngeal Carcinoma Tumor Tissues and Cell Lines

**FIGURE 1:**
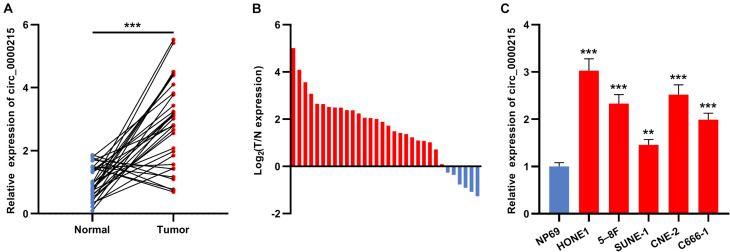
Circ_0000215 expression is up-modulated in NPC. **(A)** qRT-PCR was performed to detect circ_0000215 expression in 32 pairs of NPC tissues and normal tissues adjacent to cancer. **(B)** Circ_0000215 expression was up-modulated in 81.25% (26/32) of the 32 NPC tissues. **(C)** Circ_0000215 expression in NPC cell lines (HONE1, 5-8F, SUNE-1, CNE-2, and C666-1) and immortalized normal nasopharyngeal epithelial cell line NP69 cells was examined by qRT-PCR. The significance of difference was determined by Student’s *t*-test or one-way ANOVA. **P < 0.01, and ***P < 0.001. Data from three independent experiments were expressed as mean ± standard deviation.

First of all, circ_0000215 expression in 32 pairs of NPC and normal tissues was examined by qRT-PCR. The data revealed that circ_0000215 expression was remarkably up-modulated in NPC tissues (Figure 1A and Supplementary Figure 1). Besides, 81.25% (26/32) of the 32 NPC tissues showed increased circ_0000215 expression compared with normal tissues (Figure 1B). In addition, qRT-PCR showed that circ_0000215 was also significantly highly expressed in NPC cell lines (HONE1, 5-8F, SUNE-1, CNE-2, and C666-1) compared to immortalized normal nasopharyngeal epithelial cell line NP69 cells (Figure 1C and Supplementary Figure 1). We then analyzed the relationship between circ_0000215 expression and the clinicopathological characteristics of the patients. The results revealed that the expression of circ_0000215 in patients was correlated with lymph node metastasis and higher clinical stage of the patients (Table 1). The above data implied that circ_0000215 might exert promoting effects in NPC progression.

### Characterization of Circ_0000215 in Nasopharyngeal Carcinoma Cells

**FIGURE 2:**
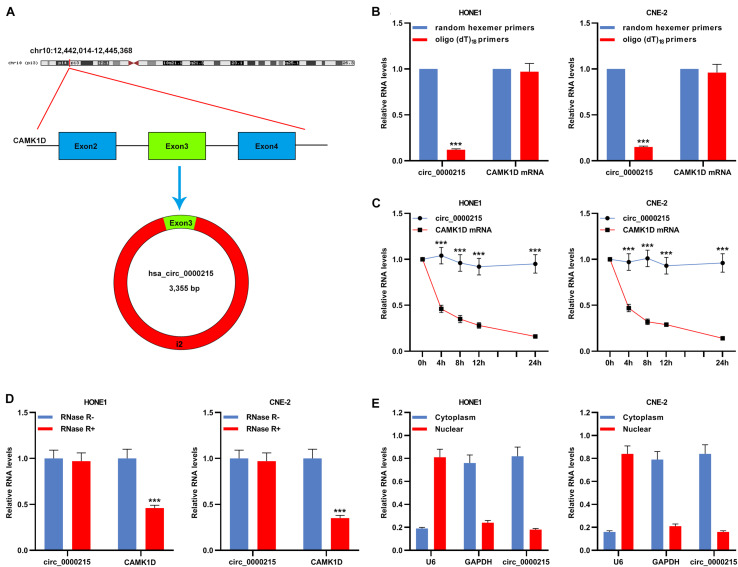
Characterization of circ_0000215. **(A)** The schematic diagram illustrated the production of circ_0000215. **(B)** Reverse transcription was carried out using random hexamer or oligo (dT)_18_ primers. Then qRT-PCR was performed to detect the relative expression levels of circ_0000215 and CAMK1D mRNA. **(C)** qRT-PCR was executed to detect the relative expression levels of circ_0000215 and CAMK1D mRNA after HONE1 and CNE-2 cells were treated with actinomycin D. **(D)** qRT-PCR was performed to detect the enrichment of circ_0000215 and CAMK1D after the total RNA was incubated with or without RNase R. **(E)** The relative expression level of circ_0000215 in the cytoplasm and nucleus of HONE1 and CNE-2 cells was detected by qRT-PCR, and GAPDH and U6 were used as positive controls of the cytoplasm and nucleus, respectively. The significance of difference was determined by Student’s *t*-test. ***P < 0.001. Data from three independent experiments were expressed as mean ± standard deviation.

According to CircBase database, circ_0000215 is a circRNA with 3355 base pairs (bp) in length formed by reverse splicing of the transcript of the CAMK1D gene located at 10p13 (Figure 2A). To verify the circular structure of circ_0000215, RNA from HONE1 and CNE-2 cells was subjected to reverse transcription using random hexamer or oligo (dT)_18_ primers. The data of qRT-PCR revealed that circ_0000215 was only detectable when random hexamer primers were used (Figure 2B), which proved that circ_0000215 did not have a poly-A tail. The expression levels of circ_0000215 and CAMK1D were measured in HONE1 and CNE-2 cells which were treated with actinomycin D to restrain transcription. The data revealed that circ_0000215 was much more stable than CAMK1D mRNA (Figure 2C and Supplementary Figure 2). Moreover, total RNA of HONE1 and CNE-2 cells was incubated with RNase R, and after that, qRT-PCR revealed that circ_0000215 was resistant to the degradation induced by RNase R, while CAMK1D mRNA was degraded by RNase R (Figure 2D and Supplementary Figure 2). Nucleoplasmic isolation experiments demonstrated that circ_0000215 was mainly localized in the cytoplasm of HONE1 and CNE-2 cells (Figure 2E).

### Knocking Down Circ_0000215 Expression Represses the Growth, Migration and Invasion of Nasopharyngeal Carcinoma Cells *in vitro*

**FIGURE 3:**
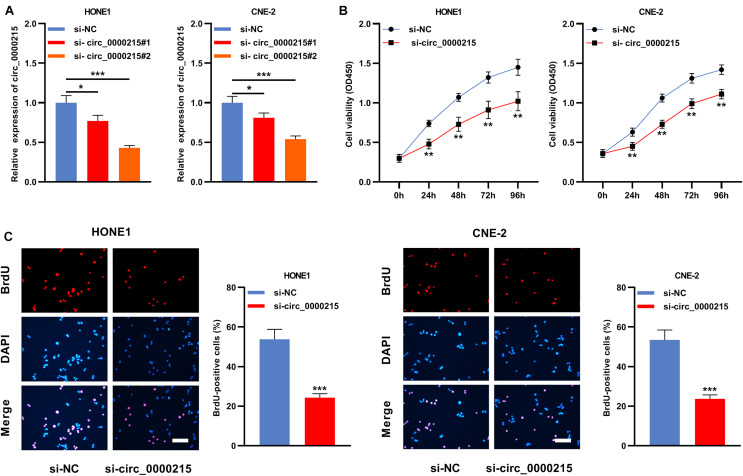
Knockdown of circ_0000215 impedes the multiplication of NPC cells. **(A)** Two siRNAs against circ_0000215 (si-circ_0000215#1 and si-circ_0000215#2) were transfected into HONE1 and CNE-2 cells, respectively. qRT-PCR confirmed that the expression of circ_0000215 was reduced after the transfection. **(B)** CCK-8 experiment was employed to detect the viability of HONE1 and CNE-2 cells transfected with si-circ_0000215. **(C)** BrdU experiment was implemented to examine the proliferation of HONE1 and CNE-2 cells transfected with si-circ_0000215. Scale bar, 50 μM. The significance of difference was determined by Student’s *t*-test or one-way ANOVA. *P < 0.05, **P < 0.01, and ***P < 0.001. Data from three independent experiments were expressed as mean ± standard deviation.

**FIGURE 4:**
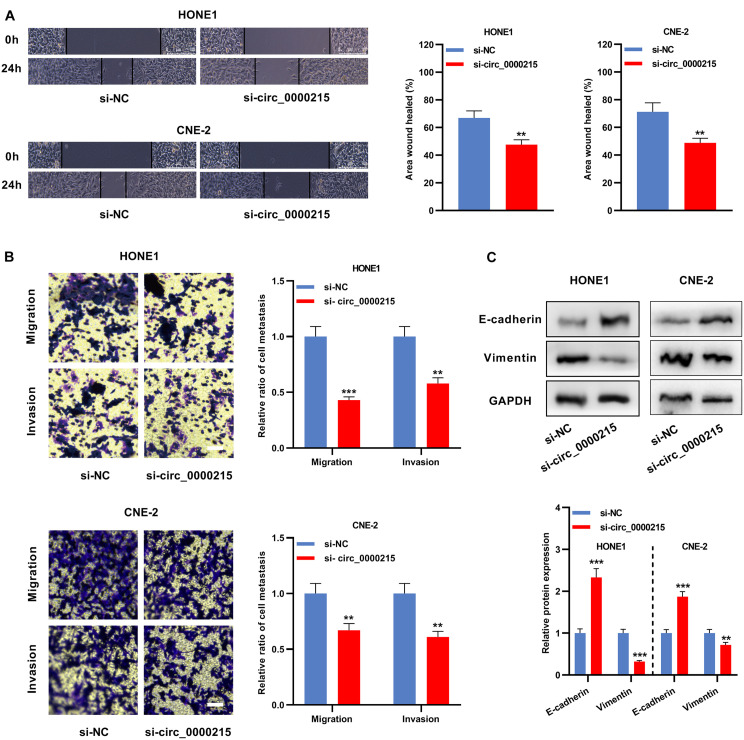
Circ_0000215 inhibition represses the metastatic potential of NPC cells. **(A)** The wound-healing experiment was carried out to detect the motility of HONE1 and CNE-2 cells transfected with si-circ_0000215. **(B)** Transwell experiment was executed to detect migration and invasion of HONE1 and CNE-2 cells transfected with si-circ_0000215. Scale bar, 50 μM. **(C)** Western blot was used to detect the expression levels of E-cadherin and Vimentin in HONE1 and CNE-2 cells transfected with si-circ_0000215. The significance of difference was determined by Student’s *t*-test. *P < 0.05, **P < 0.01, and ***P < 0.001. Data from three independent experiments were expressed as mean ± standard deviation.

Circ_0000215 showed the highest expression level in HONE1 and CNE-2 cells. Therefore, siRNAs targeting circ_0000215 (si-circ_0000215#1/2) and controls were transfected into HONE1 and CNE-2 cells. si-circ_0000215#2, with higher knockdown efficiency, was selected for subsequent experiments (Figure 3A and Supplementary Figure 3). Also, the knockdown of circ_0000215 did not affect the expression level of linear CAMK1D mRNA (Supplementary Figure 3). The data of CCK-8 experiments and BrdU assay revealed that cell viability was remarkably repressed in both HONE1 and CNE-2 cells in the si-circ_0000215 group relative to the control group (Figures 3B,C). Additionally, the data of scratch healing assay and Transwell experiment showed that migration and invasion of NPC cells was remarkably suppressed in the si-circ_0000215 group (Figures 4A,B). Western blot experiments showed that knockdown of circ_0000215 suppressed Vimentin expression, enhanced E-cadherin expression (Figure 4C).

### Circ_0000215 Sponges miR-512-5p in Nasopharyngeal Carcinoma Cells

**FIGURE 5:**
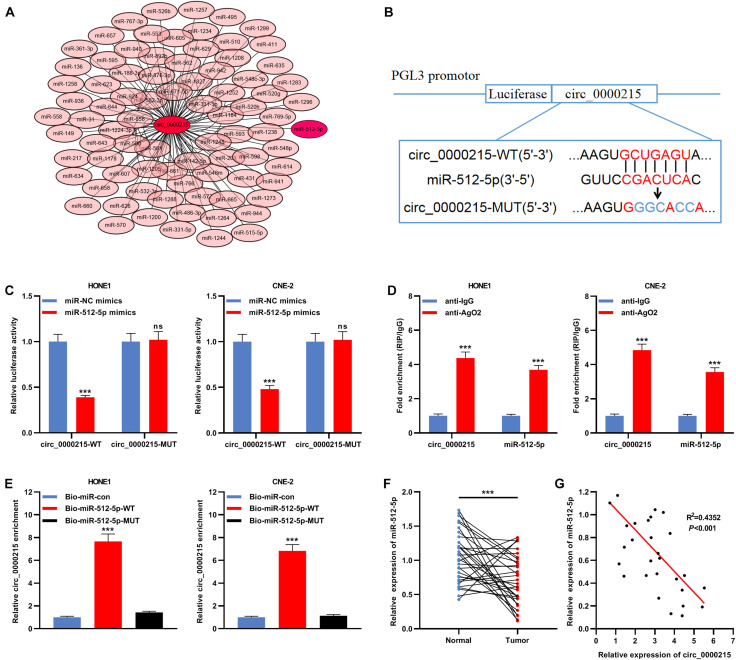
MiR-512-5p is a downstream target of circ_0000215. **(A)** Bioinformatics analysis was designed to predict the targeted miRNAs downstream of circ_0000215. **(B)** The schematic diagram showed the putative binding site between miR-512-5p and circ_0000215. **(C)** Dual-luciferase reporter gene assay was performed to validate the binding site between miR-512-5p and circ_0000215. **(D)** The complex containing circ_0000215 and miR-512-5p in HONE1 and CNE-2 cells was immunoprecipitated by anti-Ago2 in RIP assay. **(E)** RNA pull-down assay was carried out to verify the interaction between circ_0000215 and miR-512-5p in HONE1 and CNE-2 cells. **(F)** qRT-PCR was performed to detect miR-512-5p expression in 32 pairs of NPC tissues and normal tissues adjacent to the cancer. **(G)** Pearson correlation analysis was performed to analyze the correlation between circ_0000215 and miR-512-5p expressions in NPC tissues. The significance of difference was determined by Student’s *t*-test or one-way ANOVA.***P < 0.001, ns differences were not statistically significant. Data from three independent experiments were expressed as mean ± standard deviation.

The Circular RNA Interactome database was searched, and it was predicted that miR-512-5p might be the downstream target of circ_0000215 (Figure 5A and Supplementary Table 1). To elucidate the targeting relationship between circ_0000215 and miR-512-5p, circ_0000215-WT plasmid and circ_0000215-MUT plasmid containing miR-512-5p binding site were constructed for luciferase reporter gene experiments (Figure 5B). The data revealed that miR-512-5p mimics remarkably diminished the luciferase activity of circ_0000215-WT cells, while there was no remarkable effect on the luciferase activity of circ_0000215-MUT (Figure 5C). Moreover, RIP assay showed that circ_0000215 and miR-512-5p were enriched in pellets containing Ago2, but not in the pellets containing IgG (Figure 5D and Supplementary Figure 4). In HONE1 and CNE-2 cells, circ_0000215 could be pulled down by Bio-miR-512-5p-WT instead of Bio-miR-512-5p-MUT or Bio-miR-con (Figure 5E and Supplementary Figure 4). Furthermore, miR-512-5p was remarkably under-expressed in NPC tissues relative to normal tissues adjacent to the cancer (Figure 5F and Supplementary Figure 4). Additionally, Pearson’s correlation analysis revealed that miR-512-5p expression was negatively correlated with circ_0000215 expression in NPC samples (*R*^2^ = 0.4352, *P* < 0.001) (Figure 5G).

### Inhibition of miR-512-5p Expression Promotes the Growth, Migration, and Invasion of Nasopharyngeal Carcinoma Cells After Knockdown of Circ_0000215

**FIGURE 6:**
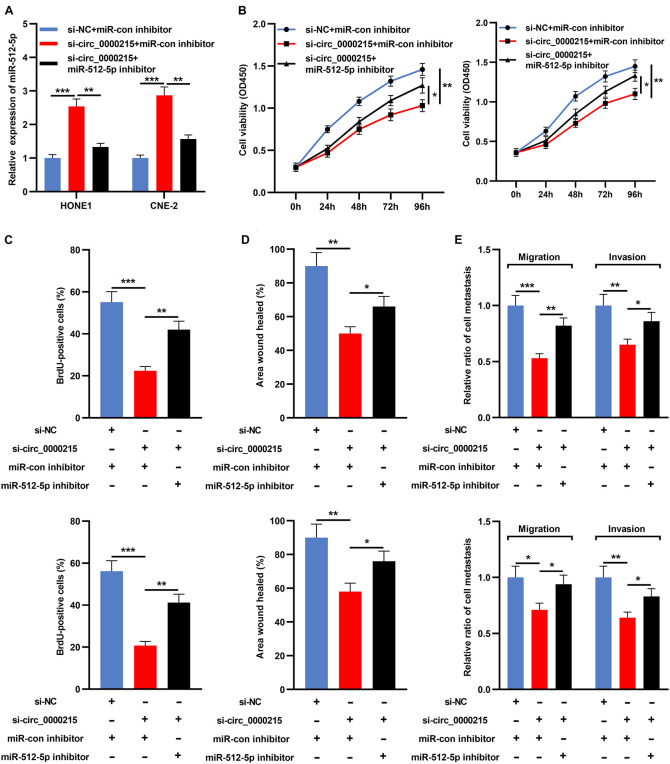
Inhibition of miR-512-5p partially reverses the inhibitory effect of circ_0000215 knockdown on NPC cells. **(A)** HONE1 and CNE-2 cells were transfected with control siRNA, si-circ_0000215 or si-circ_0000215 + miR-512-5p inhibitor, and then miR-512-5p expression was detected by qRT-PCR. **(B)** CCK-8 experiment was executed to detect the viability of HONE1 and CNE-2 cells after the transfection. **(C)** BrdU assay was employed to detect the proliferation of HONE1 and CNE-2 cells after the transfection. **(D)** Wound-healing assay was utilized to detect the motility of HONE1 and CNE-2 cells after the transfection. **(E)** Transwell assay was performed to detect the migration and invasion of HONE1 and CNE-2 cells after the transfection. The significance of difference was determined by Student’s *t*-test or one-way ANOVA.*P < 0.05, **P < 0.01, and ***P < 0.001. Data from three independent experiments were expressed as mean ± standard deviation.

Transfection of miR-512-5p inhibitor could significantly inhibit the expression of miR-512-5p (Supplementary Figure 5). Next, miR-512-5p inhibitor was co-transfected with si-circ_0000215 into HONE1 and CNE-2 cells (Figure 6A and Supplementary Figure 5). CCK-8 and BrdU assays revealed that knockdown of circ_0000215 restrained the viability and proliferation of HONE1 and CNE-2 cells and the inhibition of miR-512-5p partially counteracted these effects (Figures 6B,C). Additionally, scratch healing assay and Transwell experiments revealed that inhibition of miR-512-5p expression partially reversed the inhibitory effects of circ_0000215 knockdown on the migration and invasion of HONE1 and CNE-2 cells (Figures 6D,E).

### Circ_0000215 Modulates PIK3R1 by Sponging miR-512-5p

**FIGURE 7:**
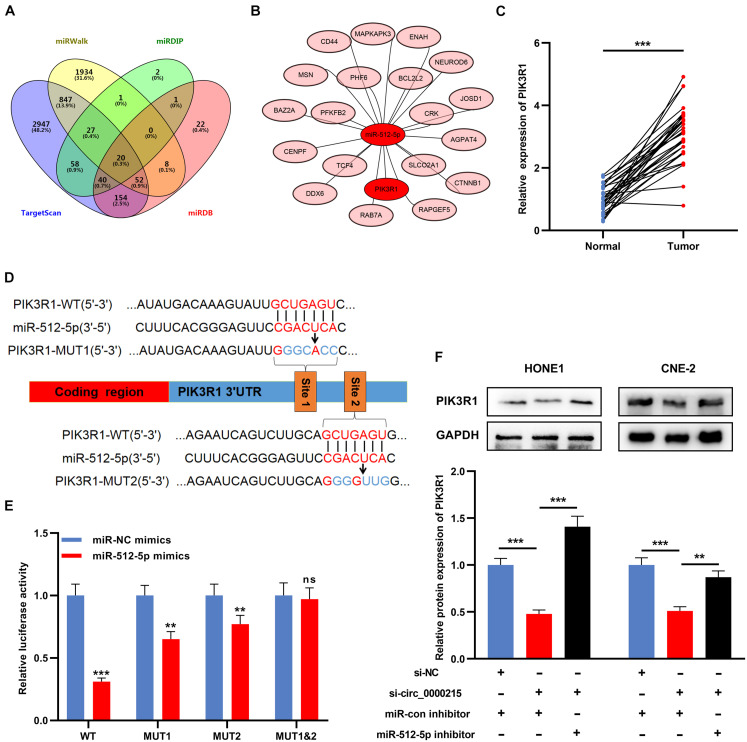
Circ_0000215 modulates PIK3R1 through sponging miR-512-5p. **(A)** Venn diagram was employed to show the targets of miR-512-5p predicted by TargetScan, miRWalk, miRDB and mirDIP. **(B)** The schematic diagram showed the predicted targets of miR-512-5p. **(C)** qRT-PCR was performed to detect PIK3R1 expression in 32 pairs of NPC tissues and normal tissues adjacent to cancer. **(D)** The schematic diagram showed the binding sites between miR-512-5p and PIK3R1 3′UTR. **(E)** Dual-luciferase reporter gene assay was performed to validate the binding sites between miR-512-5p and PIK3R1 3′UTR. **(F)** Western blot was utilized to detect the effects of circ_0000215 and miR-512-5p on PIK3R1 expression in HONE1 and CNE-2 cells. The significance of difference was determined by Student’s *t*-test or one-way ANOVA.**P < 0.01, and ***P < 0.001, ns differences were not statistically significant. Data from three independent experiments were expressed as mean ± standard deviation.

Next, bioinformatics databases (TargetScan, miRWalk, miRDB, and miRDIP) were utilized to identify potential targets of miR-512-5p, and 20 candidate target genes were obtained, including PIK3R1 (Figures 7A,B). In NPC tissue, PIK3R1 expression was higher than that in normal tissues (Figure 7C and Supplementary Figure 5). To pinpoint the targeting relationship between PIK3R1 and miR-512-5p, PIK3R1-WT reporter and PIK3R1-MUT reporter containing miR-512-5p binding site were constructed for luciferase reporter gene experiments (Figure 7D). The data showed that miR-512-5p mimic remarkably weakened the luciferase activity of PIK3R1-WT, while it exerted no remarkable effect on the luciferase activity of PIK3R1-MUT (Figure 7E). Furthermore, Western blot revealed that knockdown of circ_0000215 remarkably suppressed PIK3R1 expression, while the down-regulation of miR-512-5p partially counteracted this effect (Figure 7F).

### Circ_0000215 Enhances the Growth and Metastasis of Xenograft Tumors *in vivo*

**FIGURE 8:**
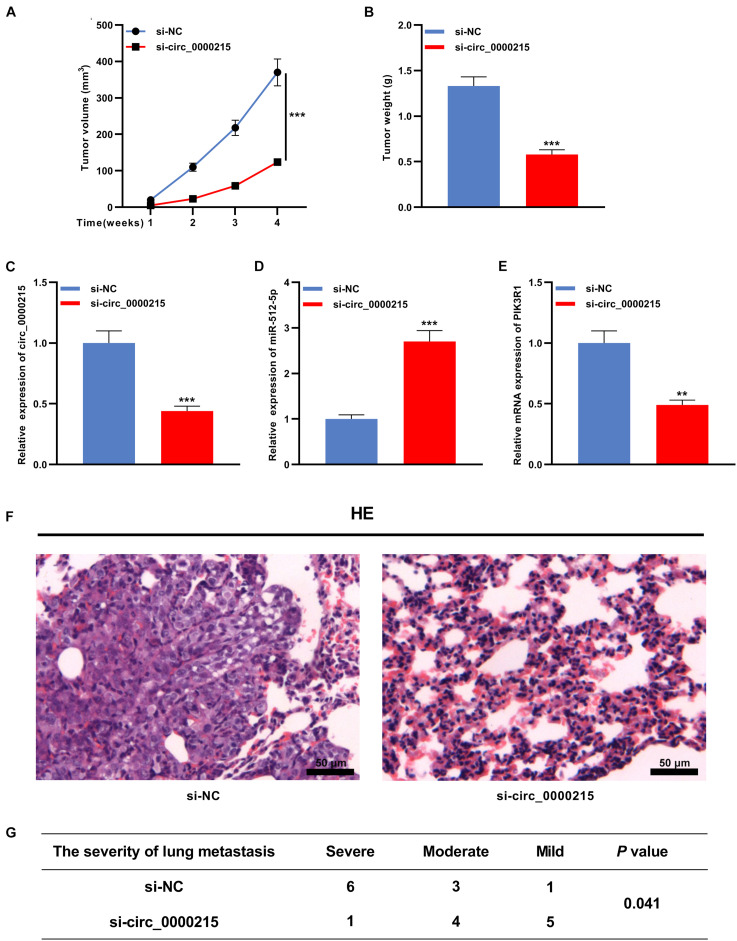
Circ_0000215 enhances the growth and lung metastasis of NPC *in vivo*. **(A,B)** HONE1 cells transfected with sh-NC or sh-circ_0000215 were injected subcutaneously into nude mice (*n* = 10 per group), and tumor growth curves **(A)** and tumor weights **(B)** were plotted. **(C–E)** qRT-PCR was employed to detect circ_0000215 **(C)**, miR-512-5p **(D)**, and PIK3R1 **(E)** expressions in tumor tissues. **(F,G)** HONE1 cells transfected with sh-NC or sh-circ_0000215 were injected into nude mice via tail vein (*n* = 10 per group), and HE staining was utilized to detect the lung metastases of the mice. The significance of difference was determined by Student’s *t*-test or chip test.**P < 0.01, and ***P < 0.001. Data from three independent experiments were expressed as mean ± standard deviation.

To confirm that circ_0000215 was indeed implicated in NPC development, a nude mouse tumor xenograft experiment was conducted. The data confirmed that the weight and volume of the tumors in the si-circ_0000215 group were lower than those in the control group (Figures 8A,B). qRT-PCR revealed that knockdown of circ_0000215 repressed circ_0000215 and PIK3R1 expressions in tumor tissues, while increasing miR-512-5p expression (Figures 8C–E and Supplementary Figure 5). Subsequently, in lung metastasis model, HE staining revealed that knockdown of circ_0000215 reduced lung metastasis of NPC cells (Figures 8F,G). These data substantiated the oncogenic role of circ_0000215 in NPC.

### Circ_0000215 Facilitates the Malignant Biological Behaviors of Nasopharyngeal Carcinoma Cells Through Activation of ERBB Signaling Pathway

**FIGURE 9:**
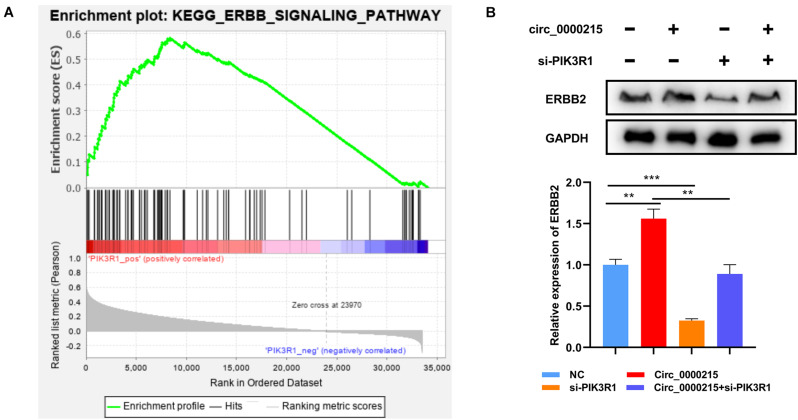
Circ_0000215 increases ERBB expression in NPC cells via PIK3R1. **(A)** GSEA revealed that PIK3R1 expression was probably associated with the ERBB signaling pathway. **(B)** Western blotting was performed to detect the effects of circ_0000215 overexpression and PIK3R1 knockdown on ERBB2 expression. The significance of difference was determined by one-way ANOVA. **P < 0.01 and ***P < 0.001. Data from three independent experiments were expressed as mean ± standard deviation.

To further elucidate the molecular mechanism of circ_0000215 modulating NPC progression, Gene Set Enrichment Analysis (GSEA) was executed using gene expression data from TCGA, and the data verified that high expression of PIK3R1 in NPC samples was positively linked to the activation of ERBB signaling (Figure 9A). Western blotting showed that ERBB2 expression was significantly elevated in HONE1 cells with circ_0000215 overexpression, and the co-transfection with si-PIK3R1 partially counteracted this effect (Figure 9B). These findings implied that circ_0000215 might facilitate the activation of ERBB signaling by modulating PIK3R1 expression in NPC.

## Discussion

Accumulating research reports that some circRNAs are associated with tumorigenesis (Wang and Fang, 2018; Zang et al., 2020). In NPC progression, circRNAs also play an important role. For instance, circ_0000285 is linked to tumor size, degree of differentiation, lymph node metastasis and distant metastasis of NPC patients, and it regulates the radiosensitivity of NPC cells (Shuai et al., 2018). Circ_0028007 promotes the growth, aggressiveness and chemoresistance of NPC cells (Qiongna et al., 2020). Circ-ABCB10 overexpression enhances NPC cell multiplication and metastasis through the up-modulation of ROCK1 expression (Duan et al., 2020). Additionally, circ-CRIM1 facilitates NPC metastasis and doxorubicin resistance by modulating FOXQ1 expression (Hong et al., 2020). Reportedly, circ_0000215 is overexpressed in gliomas and is remarkably linked to tumor size; circ_0000215 overexpression remarkably facilitates the growth, invasion and EMT of glioma cells but represses the apoptosis (Mutalifu et al., 2020). In this work, circ_0000215 expression was revealed to be up-modulated in both NPC tissues and cells. Additionally, knockdown of circ_0000215 suppressed the proliferation, migration, invasion, and EMT of NPC cells, which indicated that circ_0000215 might also exert an oncogenic effect in NPC.

Previous research has suggests that circRNAs may work as molecular sponges to decoy miRNAs and participate in tumor progression by modulating the expressions of the downstream targets of miRNAs. For example, circ-HIPK3 works as an oncogene in NPC and enhances the growth and invasion of NPC cells by suppressing the down-regulation of ELF3 expression induced by miR-4288 (Ke et al., 2019). Circ_0008450 regulates miR-577 / CXCL9 axis and thus impedes NPC cell multiplication and metastasis, and facilitates apoptosis (Wei et al., 2019). Circ_0046263 exerts an oncogenic effect in NPC via regulating miR-133a-5p/IGFBP3 axis (Yin et al., 2020). The present work demonstrated that miR-512-5p was a direct downstream target of circ_0000215. Previous studies have reported that miR-512-5p is associated with the tumorigenesis of diverse human malignancies, including NPC (Li J. et al., 2015; Dinami et al., 2017; Yang et al., 2020). For instance, miR-512-5p restrains the growth of head and neck squamous cell carcinoma by targeting hTERT (Li J. et al., 2015). In non-small cell lung cancer, miR-512-5p expression is down-modulated and miR-512-5p overexpression impedes cancer cell multiplication, migration, and invasion, and induces apoptosis by targeting ETS1 (Cao et al., 2019). In the present study, miR-512-5p was observed to be under-expressed in NPC, and inhibition of miR-512-5p partially counteracted the effects of circ_0000215 knockdown on NPC cells. Our data indicated that the biological function of circ_0000215 in NPC was partly mediated by miR-512-5p.

PI3K is a heterodimer composed of a modulatory subunit (p85) and a catalytic subunit (p110), of which p85α is encoded by PIK3R1 gene (Philp et al., 2001; Zhu et al., 2008). PIK3R1 is reported to be differentially expressed in multiple cancers and is linked to tumor progression and metastasis. Reportedly, PIK3R1 promotes the migration and invasion of breast cancer cells through modulating PI3K/AKT signaling (Miller et al., 2011). PIK3R1 expression is up-modulated in clinical tissue specimens of hepatocellular carcinoma and knockdown of PIK3R1 represses the malignant biological behaviors of cancer cells (Ai et al., 2018). Moreover, PIK3R1 also works as an oncogene in ovarian cancer and colonic cancer (Philp et al., 2001). In this work, PIK3R1 was identified to be a direct downstream target of miR-512-5p. Knockdown of circ_0000215 suppressed PIK3R1 expression, while the down-modulation of miR-512-5p expression restored its expression. These data suggested that the ceRNA network consisting of circ_0000215, miR-512-5p and PIK3R1 was involved in NPC progression.

The ERBB receptor family includes the epidermal growth factor receptor (EGFR) and ERBB1/HER1, ERBB2/HER2, ERBB3/HER3, and ERBB4/HER4. ERBB2 (Erb-B2 receptor tyrosine kinase 2, also known as HER2) attracts a lot of attention in cancer research (Soltoff et al., 1994; Schulze et al., 2005; Gutierrez and Schiff, 2011). ERBB2 modulates the proliferation and metastasis of cancer cells through the activation of PI3K/AKT and MAPK/ERK pathways (Arteaga and Engelman, 2014). ERBB2 overexpression / amplification are reported in multiple malignancies such as breast cancer, gastric cancer, esophageal cancer, endometrial cancer and so on, and its high expression often implies unfavorable prognosis of the patients; importantly, targeting ERBB2 has been applied in clinical practice to treat some cancers such as breast cancer and gastric cancer (Moasser, 2007; Wang, 2017). Recently, some studies have reported that, and targeting ERBB2 has the potential to sensitize NPC cells to chemotherapy and radiotherapy (Liu et al., 2016; Huang et al., 2019; Fu et al., 2021). In this work, bioinformatics analysis suggested that PIK3R1 was associated with the activation of ERBB2. Intriguingly, we observed that circ_0000215 overexpression enhanced ERBB2 expression in HONE1 cells via regulating PIK3R1. However, the detailed mechanism by which circ_0000215 and PIK3R1 regulate ERBB2 remains to be investigated in the following work.

Taken together, we report that circ_0000215 expression is remarkably up-modulated in NPC, and circ_0000215 regulates the malignant biological behaviors of NPC cells via miR-512-5p / PIK3R1 axis. Our study provides useful information to explain the mechanism of circ_0000215 in NPC progression, and suggests that circ_0000215 is a potential target for NPC treatment.

## Data Availability Statement

The data used to support the findings of this study are available from the corresponding author upon request.

## Ethics Statement

The studies involving human participants were reviewed and approved by this work was endorsed by the Ethics Committee of Hainan General Hospital. The patients/participants provided their written informed consent to participate in this study. The animal study was reviewed and approved by The animal experiments followed the “Guidelines for Reporting Animal Research: *In vivo* Experiments” and were endorsed by the Animal Care and Use Committee of Hainan General Hospital.

## Author Contributions

XC, ZM, and WX: study concept, study supervision, and design. JZ and XL: acquisition of data. JH and XL: analysis and interpretation of data. WX: statistical analysis. SF and WX: drafting of the manuscript. JZ: critical revision of the manuscript. All authors read the final manuscript.

## Conflict of Interest

The authors declare that the research was conducted in the absence of any commercial or financial relationships that could be construed as a potential conflict of interest.

## Publisher’s Note

All claims expressed in this article are solely those of the authors and do not necessarily represent those of their affiliated organizations, or those of the publisher, the editors and the reviewers. Any product that may be evaluated in this article, or claim that may be made by its manufacturer, is not guaranteed or endorsed by the publisher.
