# Supplementary material for: Circ_0000215 Exerts Oncogenic Function in Nasopharyngeal Carcinoma by Targeting miR-512-5p
Source: Front Cell Dev Biol. 2021 Oct 26;9:688873. doi: 10.3389/fcell.2021.688873 (PMC8577859; doi:10.3389/fcell.2021.688873)
Supplement: Supplementary file 1 [file Data_Sheet_1.pdf]

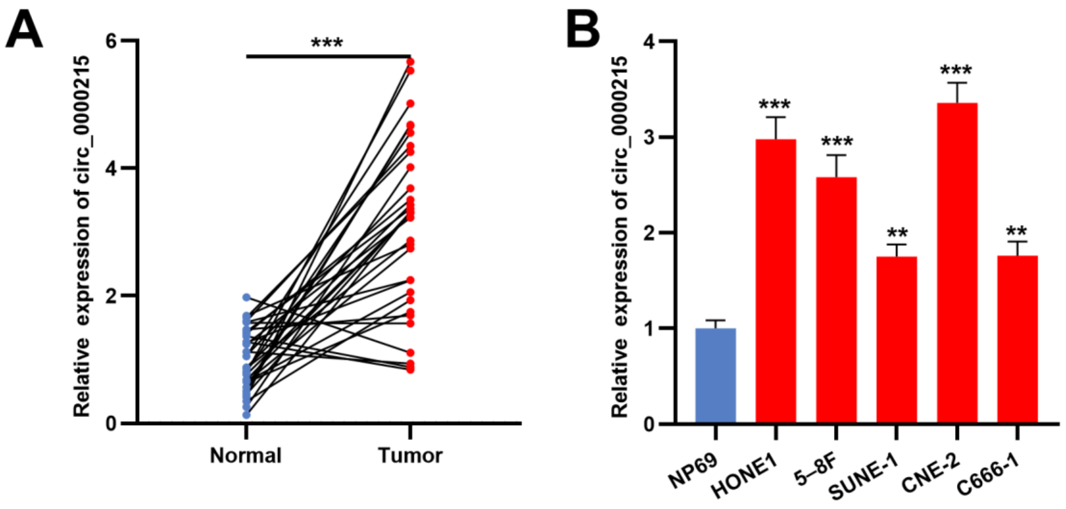

Supplementary Figure 1 qRT-PCR was performed to detect the expression of circ_0000215 in NPC tissues (A) and cells (B). β-actin was used as an endogenous control for circ_0000215. The significance was determined by Student's *t*-test or one-way ANOVA. ** $P < 0.01$, and *** $P < 0.001$. Data from three independent experiments were expressed as mean ± SD.

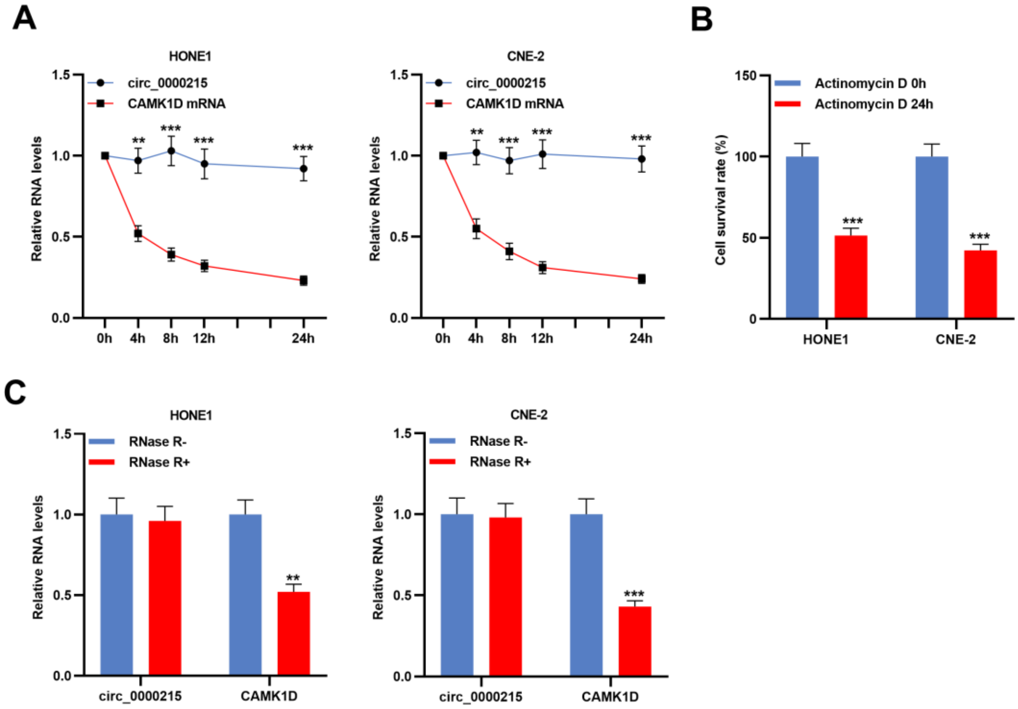

Supplementary Figure 2 qRT-PCR was performed to detect the expression of circ_0000215 and CAMK1D mRNA after HONE1 and CNE-2 cells were treated with actinomycin D (A). CCK-8 was used to detect the cell survival rate after treated with actinomycin D for 24 hours (B). qRT-PCR was performed to detect the expression of circ_0000215 and CAMK1D mRNA after the

total RNA was incubated with or without RNase R (C). β-actin was used as an endogenous control for circ_0000215 and CAMK1D. The significance was determined by Student's *t*-test or one-way ANOVA. ** *P* < 0.01, and *** *P* < 0.001. Data from three independent experiments were expressed as mean ± SD.

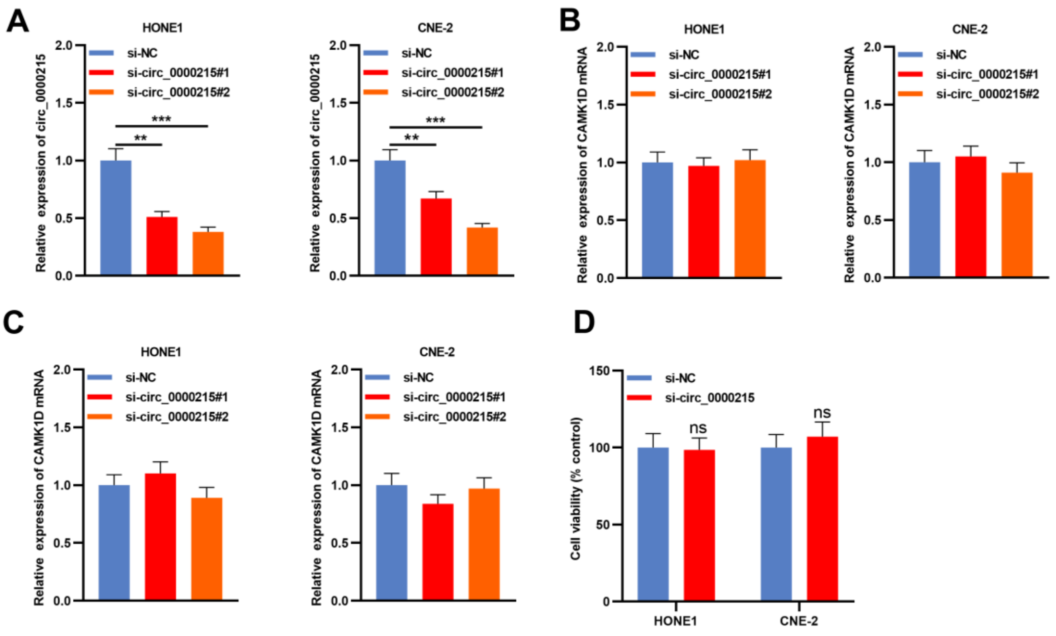

Supplementary Figure 3 qRT-PCR was performed to detect the expression of circ_0000215(A). β-actin was used as an endogenous control for circ_0000215. qRT-PCR was performed to detect the expression of CAMK1D mRNA (B-C). GAPDH was regarded as an endogenous control (B), and β-actin was regarded as an endogenous control (C). CCK-8 was used to detect the viability of cells cultured in serum-free medium for 24 hours, v.s. the cells which were cultured in complete medium (D). The significance was determined by Student's *t*-test or one-way ANOVA. ** *P* < 0.01, and *** *P* < 0.001. ns differences were not statistically significant. Data from three independent experiments were expressed as mean ± SD.

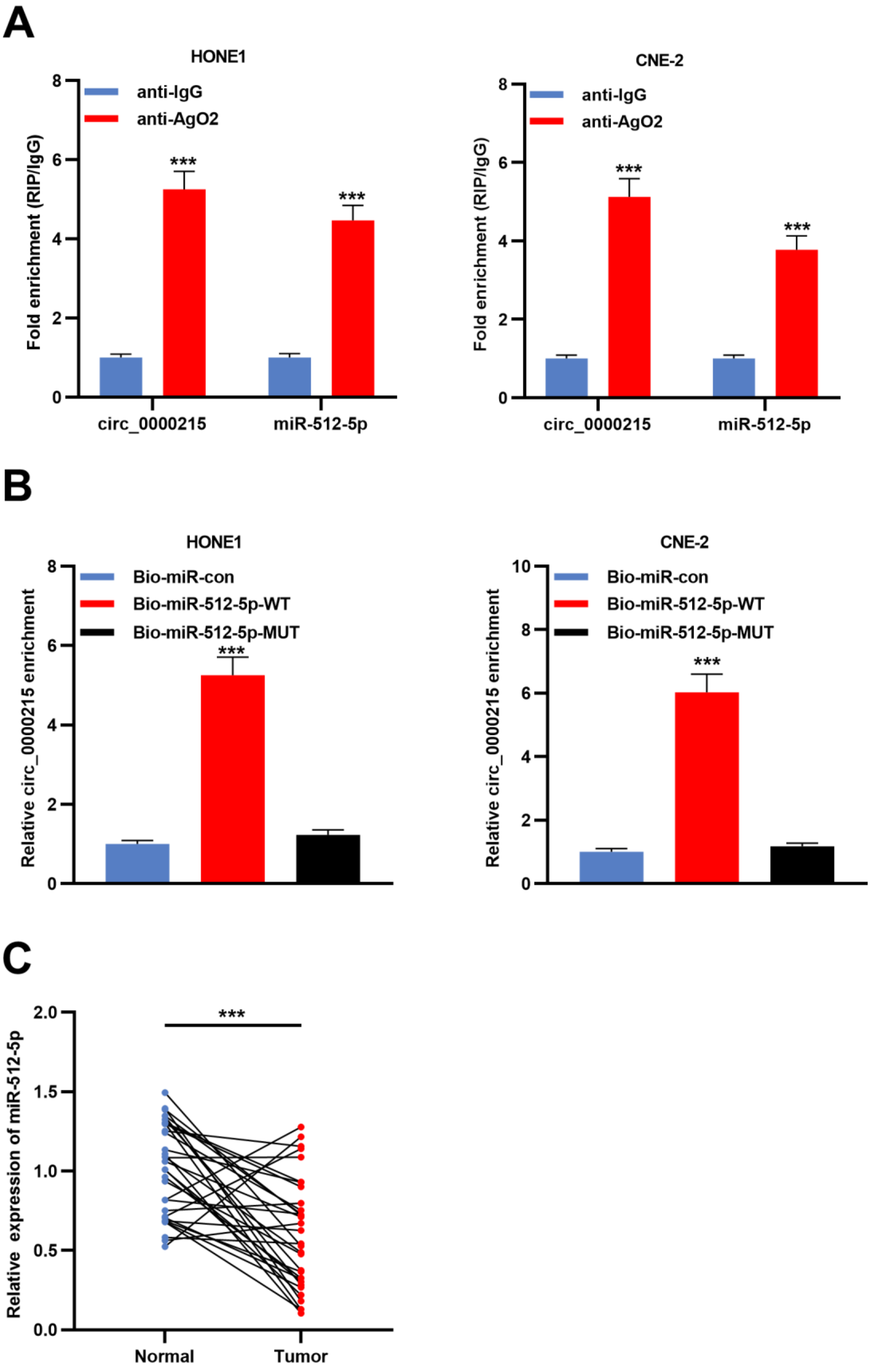

Supplementary Figure 4 qRT-PCR was performed to detect the expression of circ_0000215 and miR-512-5p. β-actin was used as an endogenous control for circ_0000215, and U48 was regarded

as the endogenous control for miR-512-5p expression levels. The significance was determined by Student's *t*-test or one-way ANOVA. *** $P < 0.001$. Data from three independent experiments were expressed as mean ± SD.

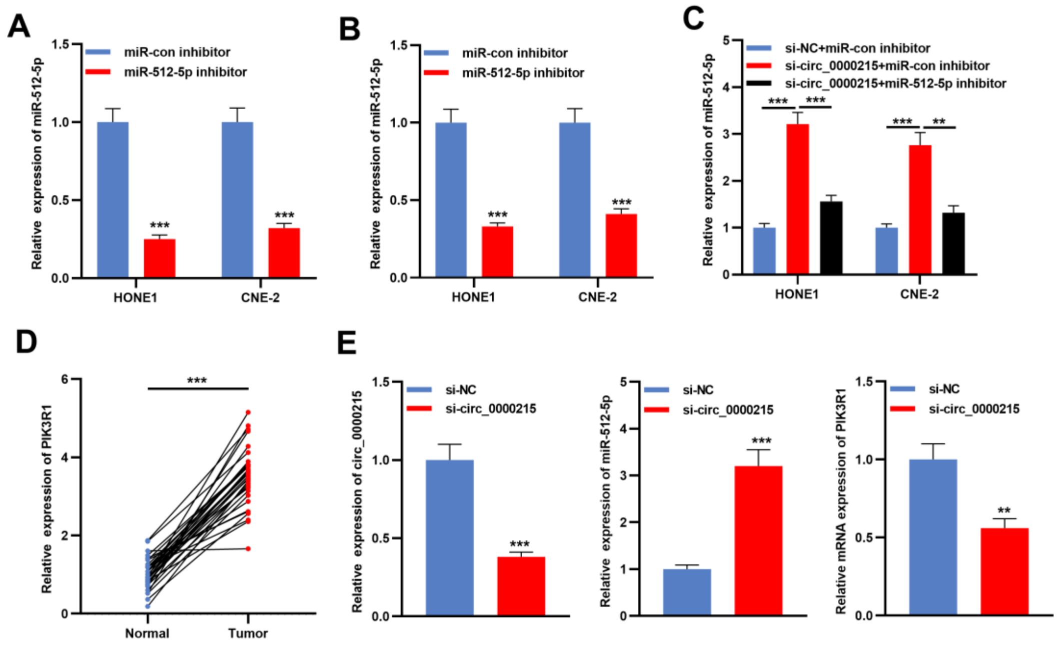

Supplementary Figure 5 qRT-PCR was performed to detect the expression of miR-512-5p (A-B). U6 and U48 was regarded as the endogenous control (A), and U48 was regarded as the endogenous control (B). qRT-PCR was performed to detect the expression of circ_0000215, miR-512-5p, and PIK3R1 expression (C-E). β-actin was used as an endogenous control for circ_0000215 and PIK3R1, and U48 was regarded as the endogenous control for miR-512-5p expression levels. The significance was determined by Student's *t*-test or one-way ANOVA. ** $P < 0.01$, and *** $P < 0.001$. Data from three independent experiments were expressed as mean ± SD.
