# Supplementary material for: Circ_0000215 Exerts Oncogenic Function in Nasopharyngeal Carcinoma by Targeting miR-512-5p
Source: Front Cell Dev Biol. 2021 Oct 26;9:688873. doi: 10.3389/fcell.2021.688873 (PMC8577859; doi:10.3389/fcell.2021.688873)

**Supplementary materials**

**Figure 2**

**HONE1 cell**

**E-cadherin**


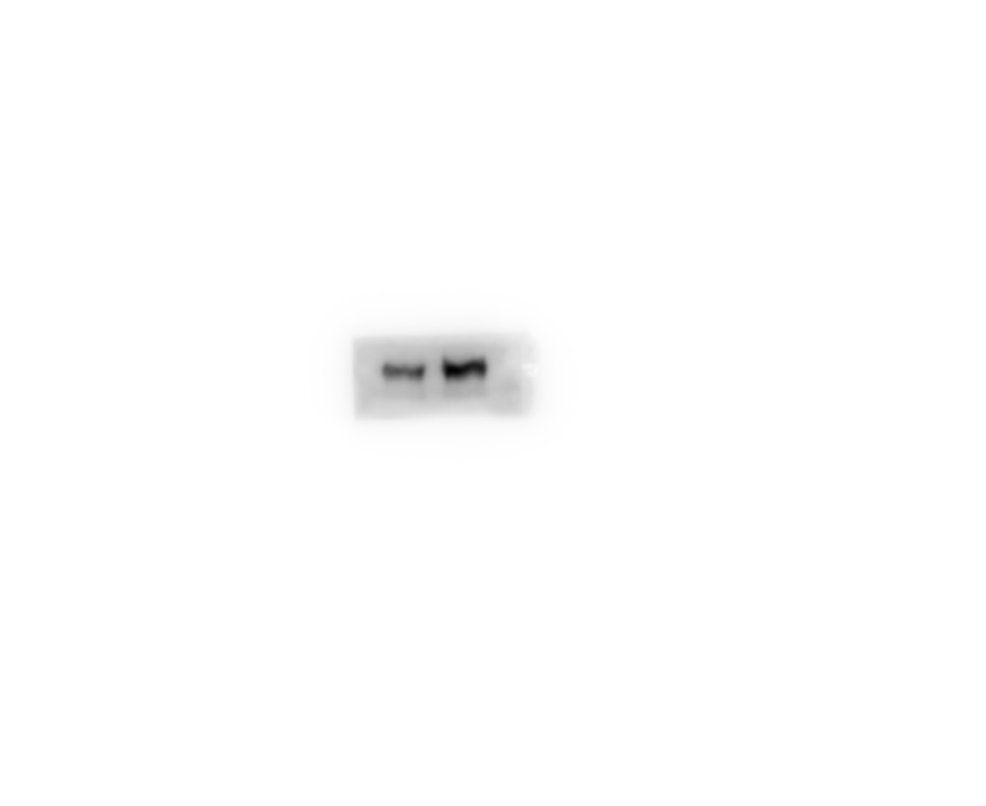


**Vimentin**


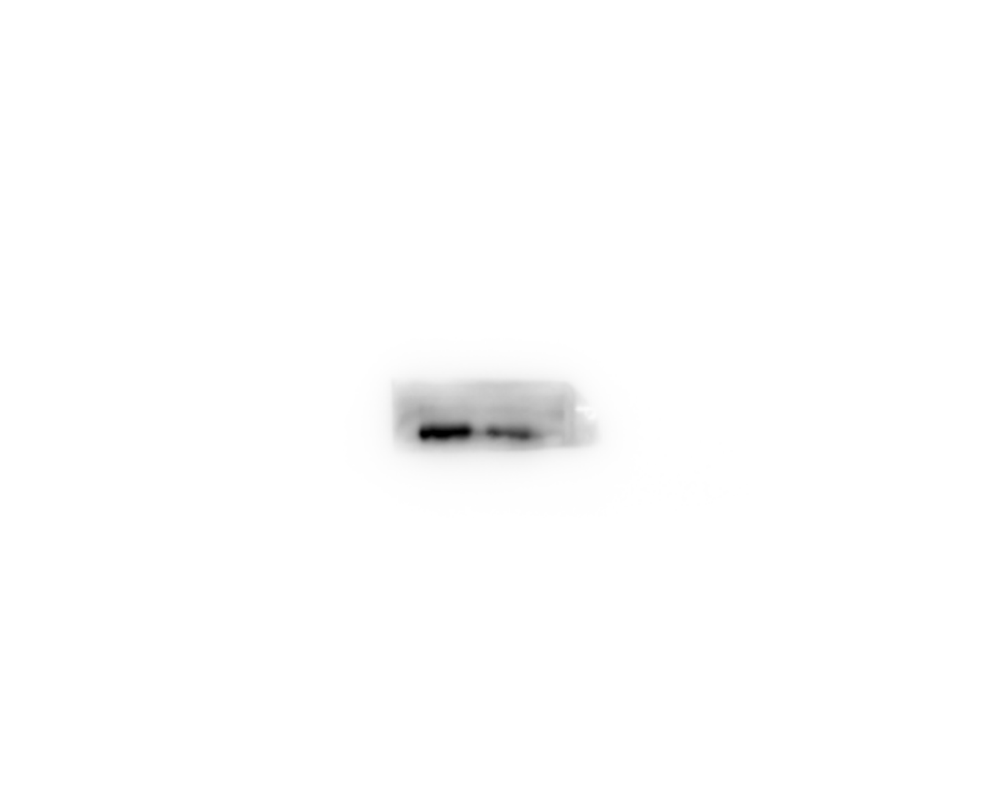


**GAPDH**


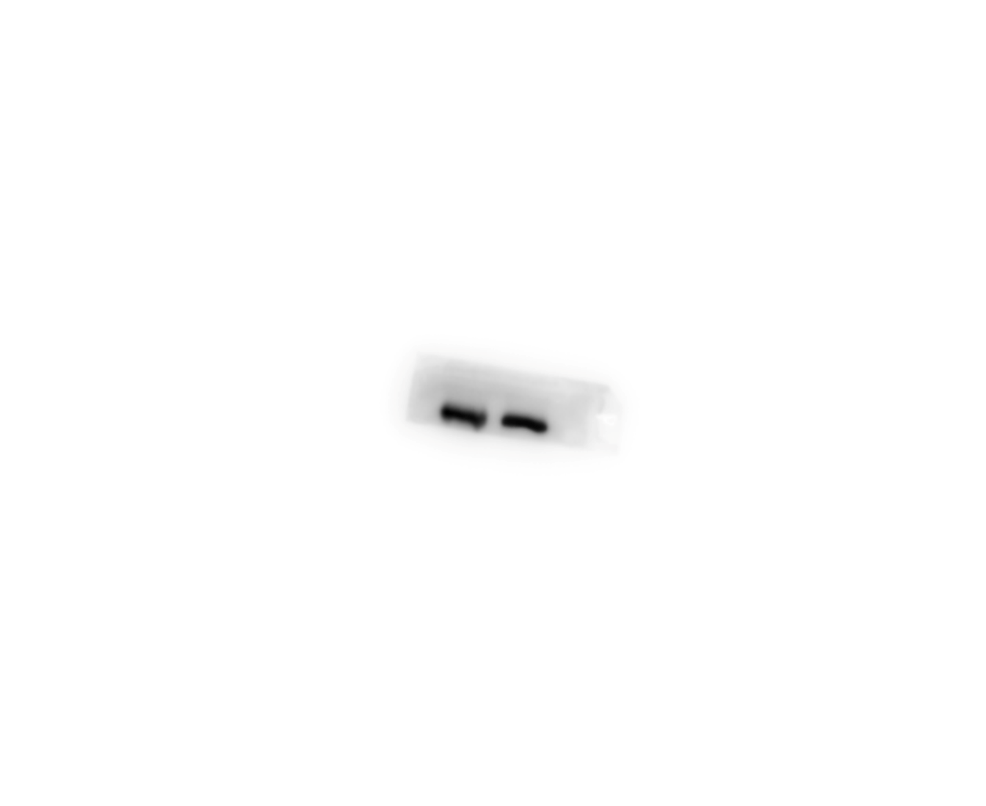


**CNE-2 cell**

**E-cadherin**


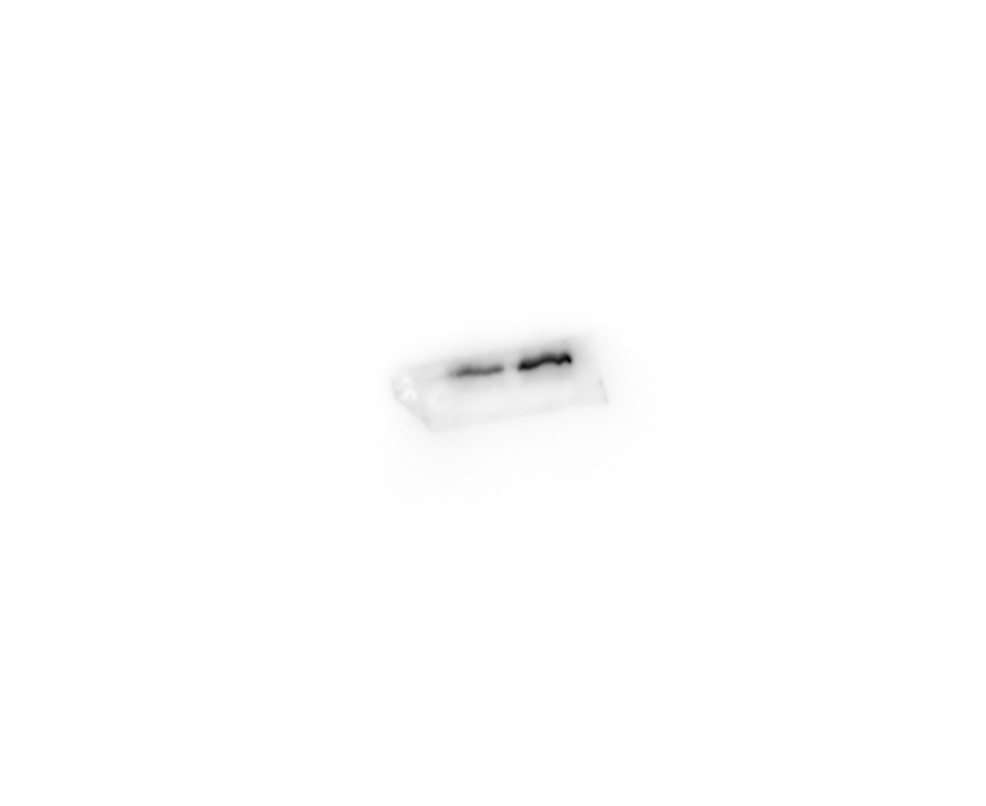


**Vimentin**


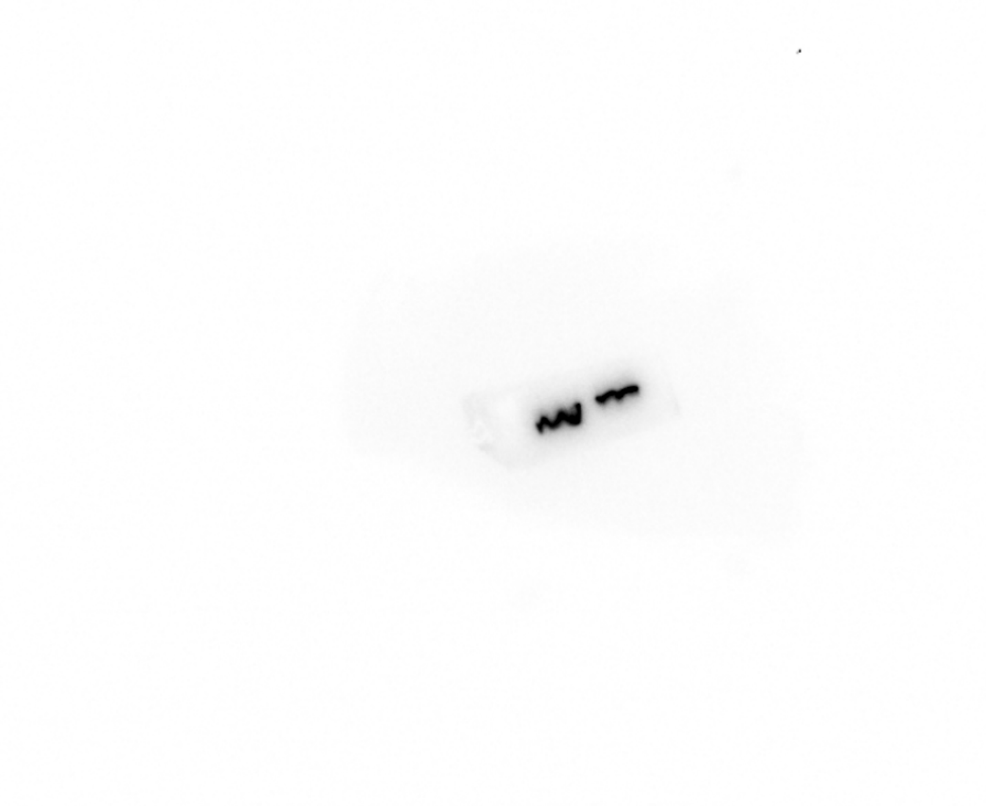


**GAPDH**


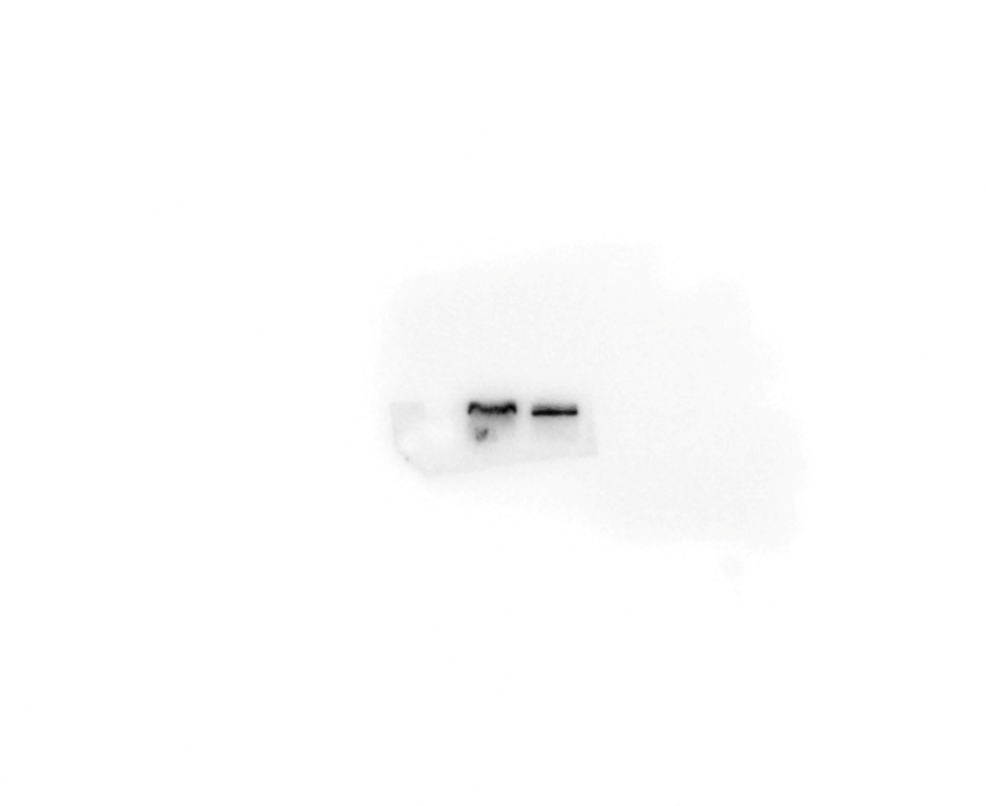


**Figure 7**

**HONE1 cell**

**PIK3R1**


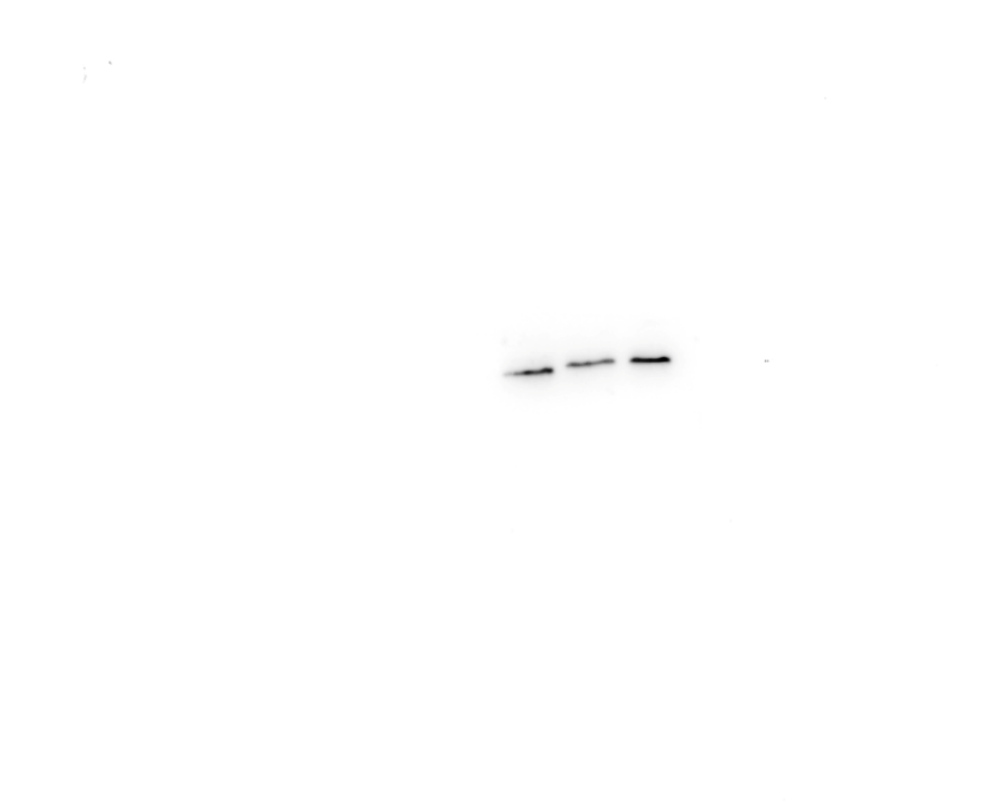


**GAPDH**


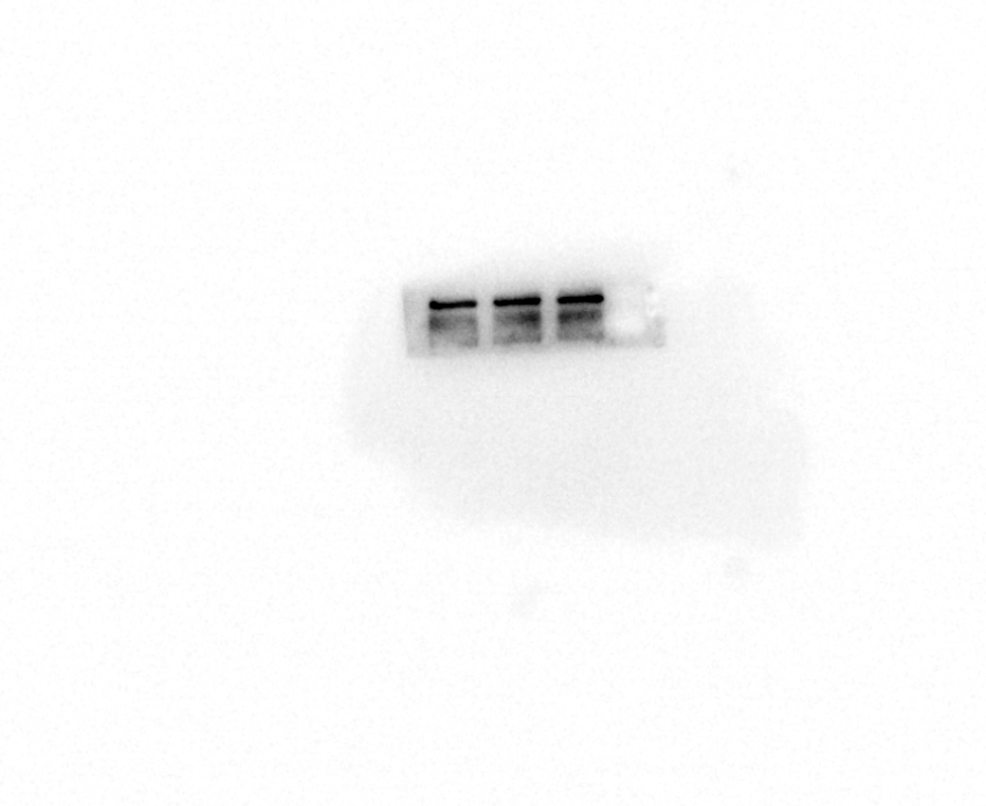


**CNE-2 cell**

**PIK3R1**


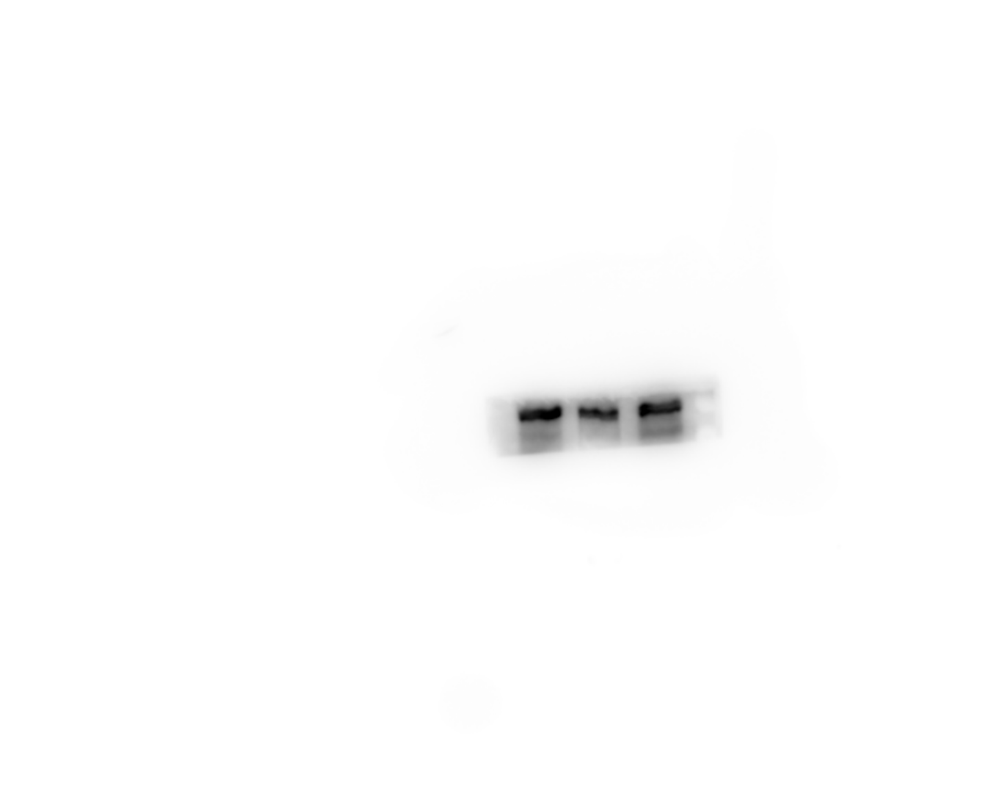


**GAPDH**


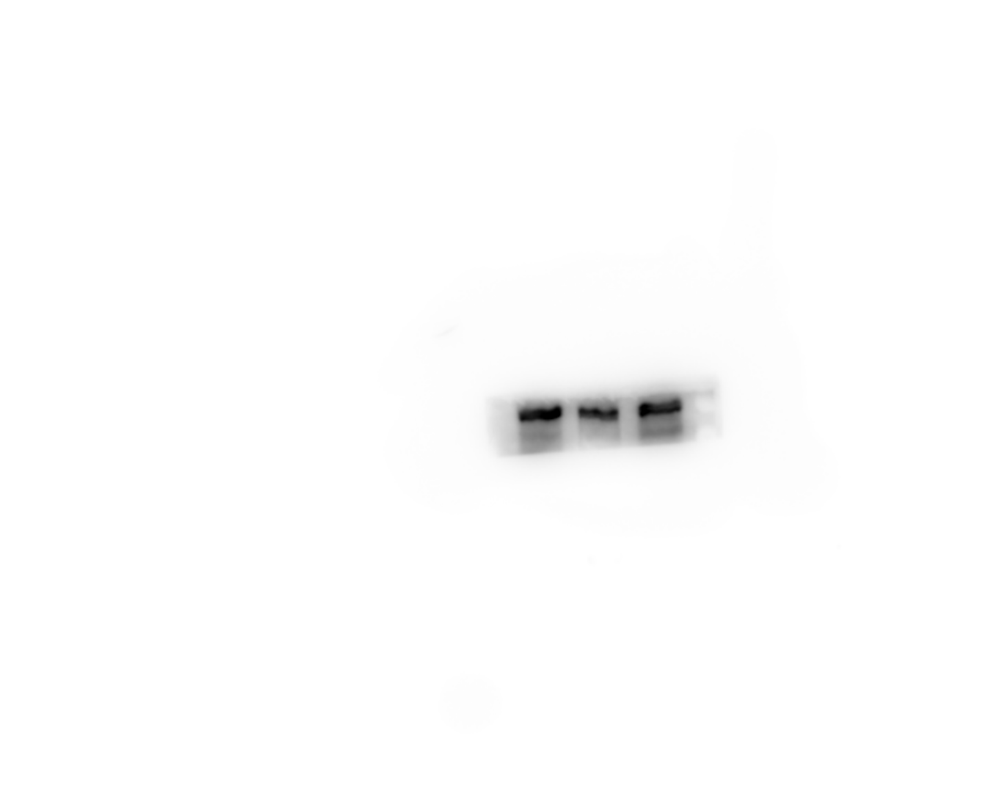


**Figure 9**

**ERBB2**


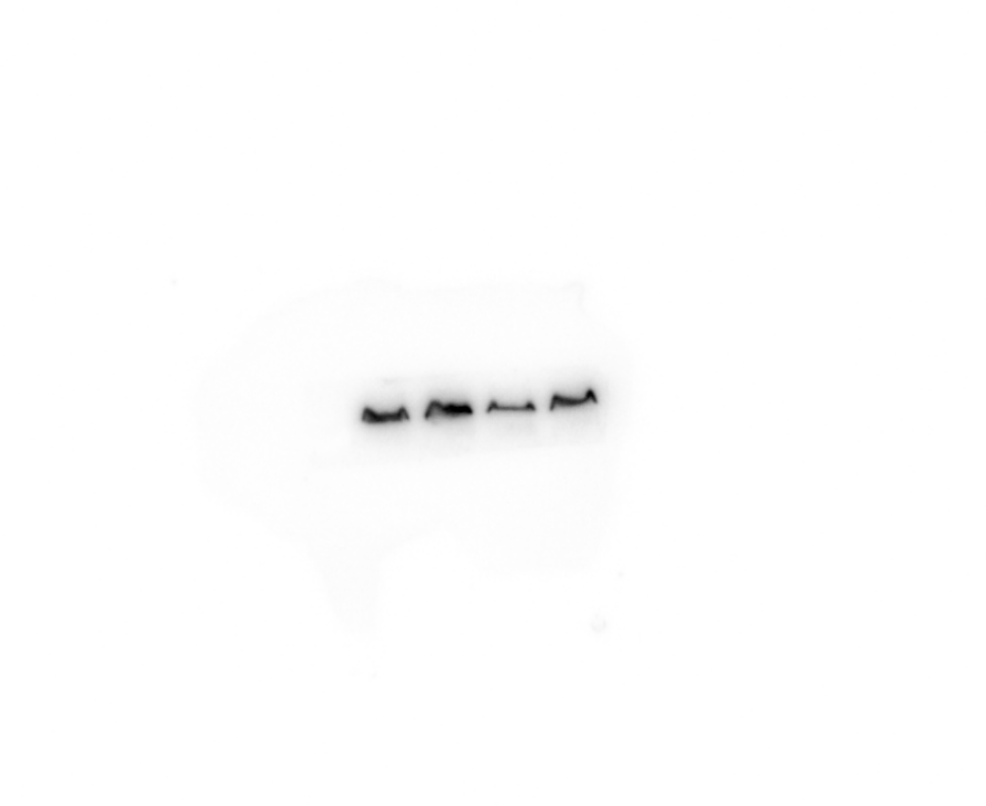


**GAPDH**


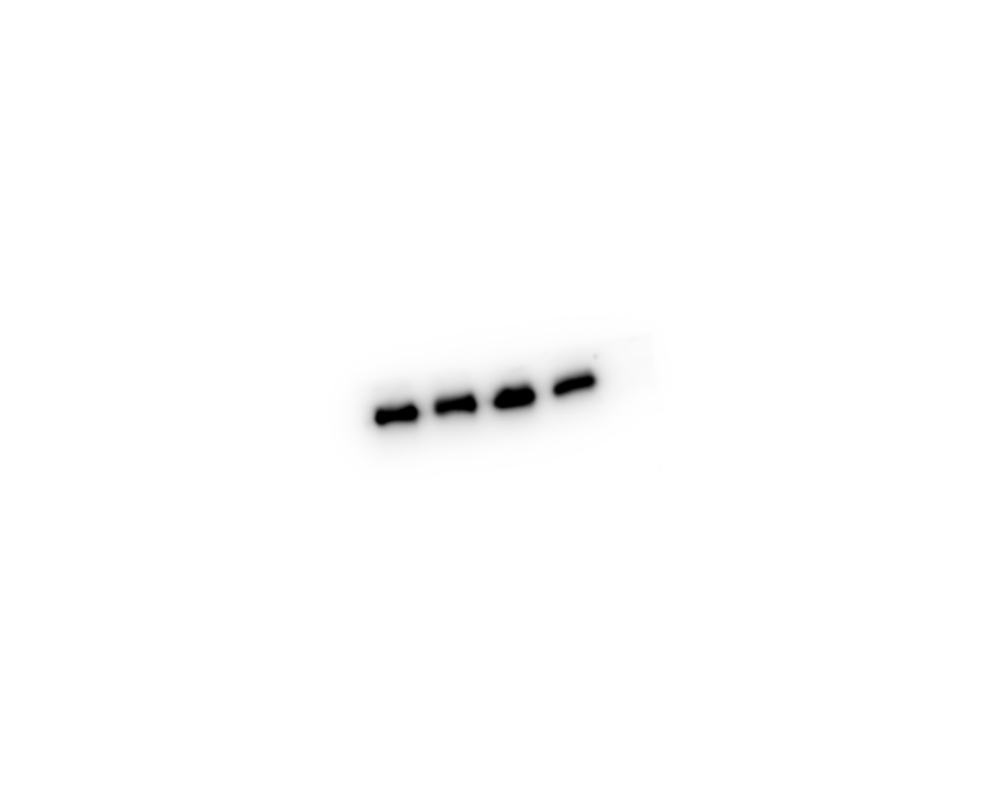

Supplement: Supplementary file 2 [file Data_Sheet_2.doc]
